# The necessity of gait evaluation on the 7th day after tap tests for the idiopathic normal pressure hydrocephalus patients

**DOI:** 10.1186/s12877-023-04481-1

**Published:** 2023-11-27

**Authors:** Ryo Oike, Yasuaki Inoue, Yoshiaki Ota, Takatoshi Sorimachi

**Affiliations:** 1 Department of Neurosurgery, Nadogaya Hospital, Chiba, Japan; 2 Division of Neuroradiology, Department of Radiology, University of Michigan, 1500 E Medical Center Dr, UH B2, Ann Arbor, MI 48109 USA; 3 Department of Neurosurgery, Tokai University, Kanagawa, Japan

**Keywords:** Timed Up Go test, Shunt surgery, Probable idiopathic normal pressure hydrocephalus, Definite idiopathic normal pressure hydrocephalus

## Abstract

**Background:**

A tap test is established as an evaluation method to indicate shunt surgery for hydrocephalus, especially idiopathic normal pressure hydrocephalus (iNPH). The timing of gait assessment after the test is still controversial, while some studies reported the effectiveness of the gait evaluation up to 2nd day after tap tests. Our study explored whether the gait evaluation on the 7th day after a tap test is necessary.

**Methods:**

We retrospectively evaluated 129 consecutive cases with possible iNPH who performed gait assessment on all 1st, 3rd, and 7th days after tap tests between May 2020 and February 2022. We reviewed the following items of the patients: age, sex, modified Rankin scale, iNPH grading scale, Mini-Mental State Evaluation (MMSE), and neurological imaging. The number of probable iNPH patients who improved their gait each day after the test was analyzed. We also assessed the number of definite iNPH patients and revealed the background characteristics of the patients who showed gait improvement on the 7th day after the tests.

**Results:**

Of the 129 patients who met our inclusion criteria, 57 were judged as probable iNPH on the 1st day, 28 were new on the 3rd, and 23 were new on the 7th. The number of probable iNPH patients up to the 7th day after tests was significantly more extensive than that of those up to the 3rd (108 [83.7%] vs. 85 [65.9%]; 95% confidence interval [CI], *p* < 0.0001). The number of definite iNPH patients was also significantly more prominent when the evaluation after the tests was performed on all of the 1st, the 3rd, and the 7th days than just on the 1st (72 vs. 42; 95% CI, *p* = 0.00016) or both of the 1st and the 3rd (72 vs. 61; 95% CI, *p* = 0.00074). No statistically significant difference existed in the patients’ backgrounds except for the pre-tap test MMSE.

**Conclusion:**

Gait evaluation on the 7th day after tap tests, in addition to the first few days, may reduce the number of iNPH patients who miss the opportunity of getting beneficial treatment.

## Introduction

Idiopathic normal pressure hydrocephalus (iNPH) is an adult-onset disease with enlarged ventricles of unknown etiology. It is characterized by the classical Hakim’s triad of gait disturbance, cognitive impairment, and urinary incontinence, which are expected to be improved by shunt surgery [1, 2]. The effect of shunt surgeries can be predicted by a tap test, widely used to determine the indication of surgical treatment [3, 4]. In a tap test, 30–50 ml of the cerebrospinal fluid (CSF) is withdrawn, and then the patient is evaluated before and after to determine if the symptoms are improved. There have been only a limited number of reports on the duration of symptomatic follow-up after tap tests. Matsuoka et al. reported that cognitive function should be measured up to 7 days after the tap test [5], and Schniepp et al. reported that the best time to evaluate gait improvement could be 2 days after the test [6]. In our institutional protocol, the result of a tap test is marked as positive if the patient experiences certain improvement in any of the clinical evaluations on the 1st, 3rd, and 7th days after the test. We have frequently experienced patients whose gait is not improved 1 or 3 days after the tap test but is improved only seven days after. We conducted this study to determine the necessity of checking the gait on the 7th day after tap tests.

## Materials and methods

### Patient population

This study was approved by the clinical research ethics reviews committee of our institution. All patients provided prior written informed consent for their participation in the study. We retrospectively evaluated 137 consecutive cases between May 2020 and February 2022 with possible iNPH, which was diagnosed if the patient met all the following features; (1) the symptoms started at the age of 60 or later, (2) at least one symptom of Hakim’s triad present: gait disturbance, cognitive impairment, or urinary incontinence, (3) brain CT or MRI demonstrates ventricular dilation, (4) no other disease can explain the symptoms, and (5) the ventricular dilation cannot be explained by other conditions, such as subarachnoid hemorrhage, meningitis, head injury, congenital hydrocephalus, and aqueductal stenosis [7]. Any symptom of the triad was considered to be present based on the episodes provided by the patient or the family member. We excluded three bed-ridden patients, four patients not evaluated per protocol, and one patient hospitalized due to a urinary tract infection 2 days after the tap test. A tap test was performed for the patients with possible iNPH within 1 month after the initial visit. One hundred twenty-nine possible iNPH cases were consequently included in this study.

### Examination of clinical factors

We retrospectively reviewed the following items from the medical records of all the enrolled patients; the age, the sex, the modified Rankin scale (mRS), the iNPH grading scale (iNPH GS), and the Mini-Mental State Evaluation (MMSE). The iNPH GS was utilized to assess the severity of Hakim’s triad using the 5-point scale from 0 to 4 [8].

### Imaging acquisition and assessment

We also reviewed the computed tomography (CT) or magnetic resonance imaging (MRI) of all the included patients to evaluate the Evans’ index (EI), the Callosal Angle (CA), the size of the temporal horn [9, 10], and the presence of Disproportionately Enlarged Subarachnoid Space Hydrocephalus (DESH) [11]. We diagnosed DESH with ventriculomegaly combined with tight sulci in high convexity and disproportionally enlarged Sylvian cisterns [11]. Each case was evaluated by two independent raters, one board-certified neurosurgeon (R.O.) with eight years of experience and one board-certified radiologist (Y.O.) with eight years of experience, blinded to the tap test result. CT scans were obtained in all 129 patients. The axial and coronal slices were used with a slice thickness of 4 mm. MRIs were obtained in the 125 patients except for 4 with pacemakers, utilizing a 1.5-T superconductive system. The T1-weighted, T2-weighted, and fluid-attenuated inversion recovery sequences were used in axial and coronal slices with a thickness of 5 mm.

### Tap test

Tap tests were performed with the paramedian puncture technique, using a 19-gauge spinal tap needle to collect 30 ml of CSF [7]. The gait was assessed using the Timed Up and Go Test (TUG) before and on the 1st, 3rd, and 7th days after the spinal tap. This test was conducted by an examiner who counts the seconds needed for the patient to stand up from a chair, walk 3 m at their usual pace, turn around a safety cone on the floor, walk back to the chair, and sit down again with their back against it [12]. We also assessed the instrumental TUG (iTUG) score, which is widely recognized for its effectiveness in quantitatively assessing mobility [13]. A positive result of the tap test was documented if the time of the TUG improved by 5 s or more or if the patients had a favorable TUG time before the test, like 15 s or less, and then their iTUG score improved by 10 points or more even when the improvement in the time was less than 5 s [7, 14].

A TUG for the patients with the mRS score of 3 was performed with their usual cane, and for those with a score of 4 was with the support of the same examiner each time. The settings of the TUGs were always identical for each patient.

### Definition of probable and definite iNPH

Probable iNPH was defined for patients with possible iNPH when their gait function improved in the tap test or when the brain imaging demonstrated the findings of DESH. Definite iNPH was defined when their gait function improved after a shunt surgery [7]. All the patients who underwent shunt surgery were seen in the clinic until at least 3 months after the surgery.

### Surgery

The shunt surgery was performed for the probable iNPH patients within 1 month after diagnosis of probable iNPH. A lumboperitoneal shunt (LPS) was selected as a first-line surgery. A ventriculoperitoneal shunt (VPS) was performed for patients with a lumbar spinal disorder, and a ventriculoarterial shunt (VAS) was performed for the patients who had a history of laparotomy with the advice by the surgeon to avoid placing the distal shunt catheter into the abdominal cavity. Codman-Certas Plus programmable valves (CSF shunt valve, Integra LifeSciences Holdings Corporation, Plainsboro Township, New Zealand) were implanted in all patients.

### Statistical analysis

The number of patients diagnosed with probable iNPH on the 1st, 3rd, and 7th days after tap tests were respectively compared by Pearson’s chi-square test. The number of definite iNPH patients was also compared by Pearson’s chi-square test among the three groups above. The age, the pre-tap test TUG, the pre-tap test MMSE, the EI, the CA, the iNPH GS, and the size of the temporal horn were compared by Mann-Whitney *U*-test between the group who showed the gait improvement on either of the 1st or the 3rd day after tap tests and the one who showed improvement only on the 7th day. Sex and the presence of DESH were compared between the two groups by the Fisher exact test. All continuous variables were described as medians (interquartile range [IQR]). All the *p*-values were two-sided, and the *p*-values of 0.05 or less were considered statistically significant. Intraclass correlation coefficients (ICC) were calculated to establish inter-rater agreement among the reviewers. Regarding the imaging assessment, ICC was used to assess the inter-observer agreement for the EI, the CA, the size of the temporal horn, and the presence of DESH. Inter-reader agreement for these contents was assessed by kappa coefficient, which was interpreted as follows: < 0.40, poor-to-fair agreement; 0.41–0.60, moderate agreement; 0.61–0.80, substantial agreement; and 0.81-1.00, almost perfect agreement. All statistical calculations were conducted using R software (version 4.1.1; R Core Team, Vienna, Austria).

## Results

**Fig. 1:**
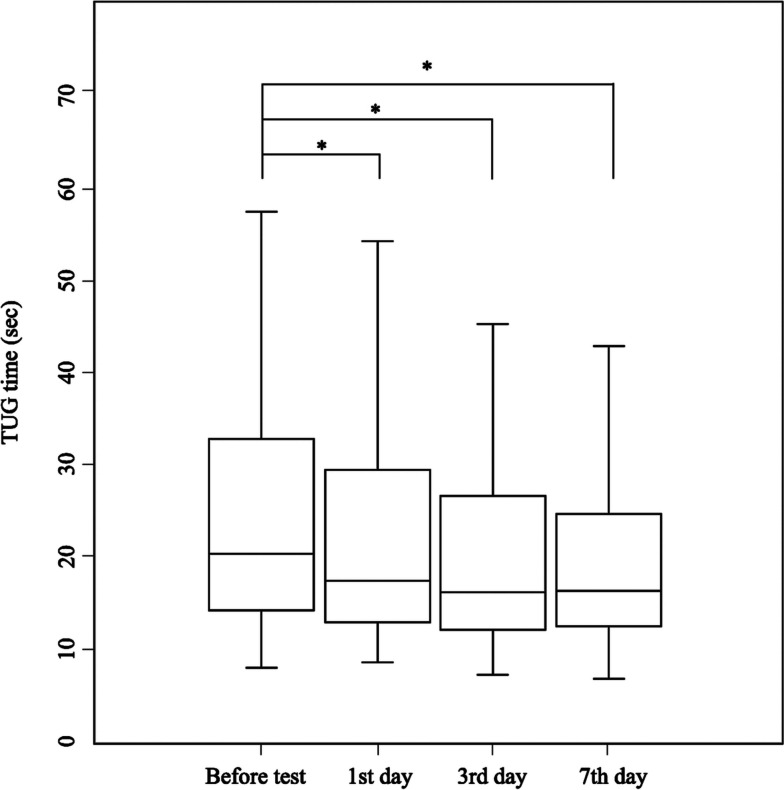
The changes in the quantitative data of the TUG time.  Wilcoxson sign-rank tests revealed that the TUG time on the 1st, 3rd, and 7th days was significantly shorter than before the test. *P* value less than 0.05 was considered significant

Our cohort comprised 129 patients. The median age was 79 (IQR, 76 to 83) (Table 1). The number of patients who showed gait improvement on any of the 1st and the 3rd days after tap tests was 85 (65.9%), while the number of those with gait improvement only on the 7th day was 23 (17.8%). Out of 85 probable iNPH who were diagnosed either on the 1st or 3rd day, 61 patients showed improvement by 5 s or more in the TUG, while out of 23 probable iNPH diagnosed only on the 7th day, 17 patients showed this improvement. The changes in quantitative TUG time data are shown in Fig. 1.

**Table 1:** Background characteristics of the probable iNPH patients who underwent tap tests

Variable	Overall (*n*=108)	1st or 3rd day^a^ (*n* = 85)	7th day^b^ (*n* = 23)	*p* value
**Demography**				
Age, median (IQR)	79.5 (76–83)	79 (76–83)	81 (77−83.5)	0.51
Male, n (%)	59 (54.6)	48 (56.5)	11 (47.8)	0.49
**iNPH grading scale**				
Gait (0–4), median (IQR)	2 (1–3)	2 (1–3)	2 (1.5−3)	0.75
Cognition (0–4), median (IQR)	2 (1–3)	2 (1–3)	3 (1.5−3)	0.24
Urinary (0–4), median (IQR)	2 (1–2)	2 (1–2)	2 (1.0–3)	0.38
**Pre-tap test TUG, sec. (IQR)**	21.1 (14.7–40.4)	21.5 (14.7–40.0)	20.5 (14.6–36.0)	0.85
**Pre-tap test MMSE (IQR)**	23 (19–27)	23 (19–27)	21 (18–24)	0.044
**Image findings**				
Evans’ index, median (IQR)	0.33 (0.32–0.35)	0.33 (0.32–0.35)	0.33 (0.32–0.35)	0.85
Callosal angle, median (IQR)	84.7 (71.4–98.8)	88.0 (71.4−102.7)	80.9 (66.0−91.6)	0.17
Size of TH, median (IQR)	5.35 (4.18–6.85)	4.88 (3.68–7.15)	5.2 (4.25–6.40)	0.96
DESH, n (%)	84 (77.8)	66 (77.6)	18 (78.3)	0.95

*Abbreviations: IQR *Interquartile range, *iNPH *Idiopathic normal pressure hydrocephalus, *TUG *Timed Up Go test, *MMSE *Mini-Mental State Examination, *TH *Temporal horn, *DESH *Disproportionately enlarged subarachnoid space

*p* value less than 0.05 in the univariate analysis is considered significant

^a^The group of patients who showed gait improvement either of the 1st or 3rd day after tap tests

^b^The group of patients who showed gait improvement for the first time on the 7th day after tap tests

### Proportion of probable iNPH

**Fig. 2:**
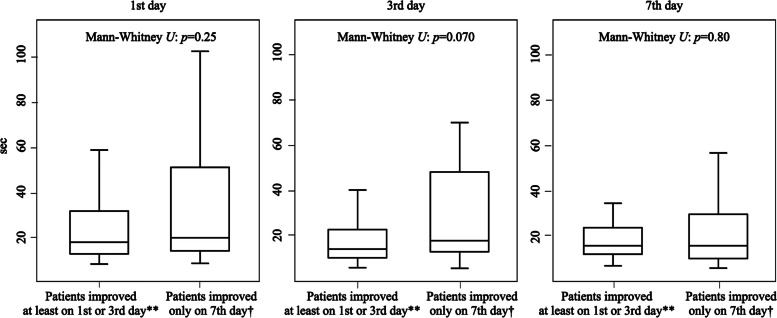
TUG Time Comparison: Gait Improvement on 1st/3rd vs. 7th day during Evaluations.  No significant difference between the two groups was shown by the Mann-Whitney U tests. *P* value less than 0.05 was considered significant. ** The group of patients who showed gait improvement either on the 1st or 3rd day after tap tests. † The group of patients who showed gait improvement for the first time on the 7th day after tap tests

**Fig. 3:**
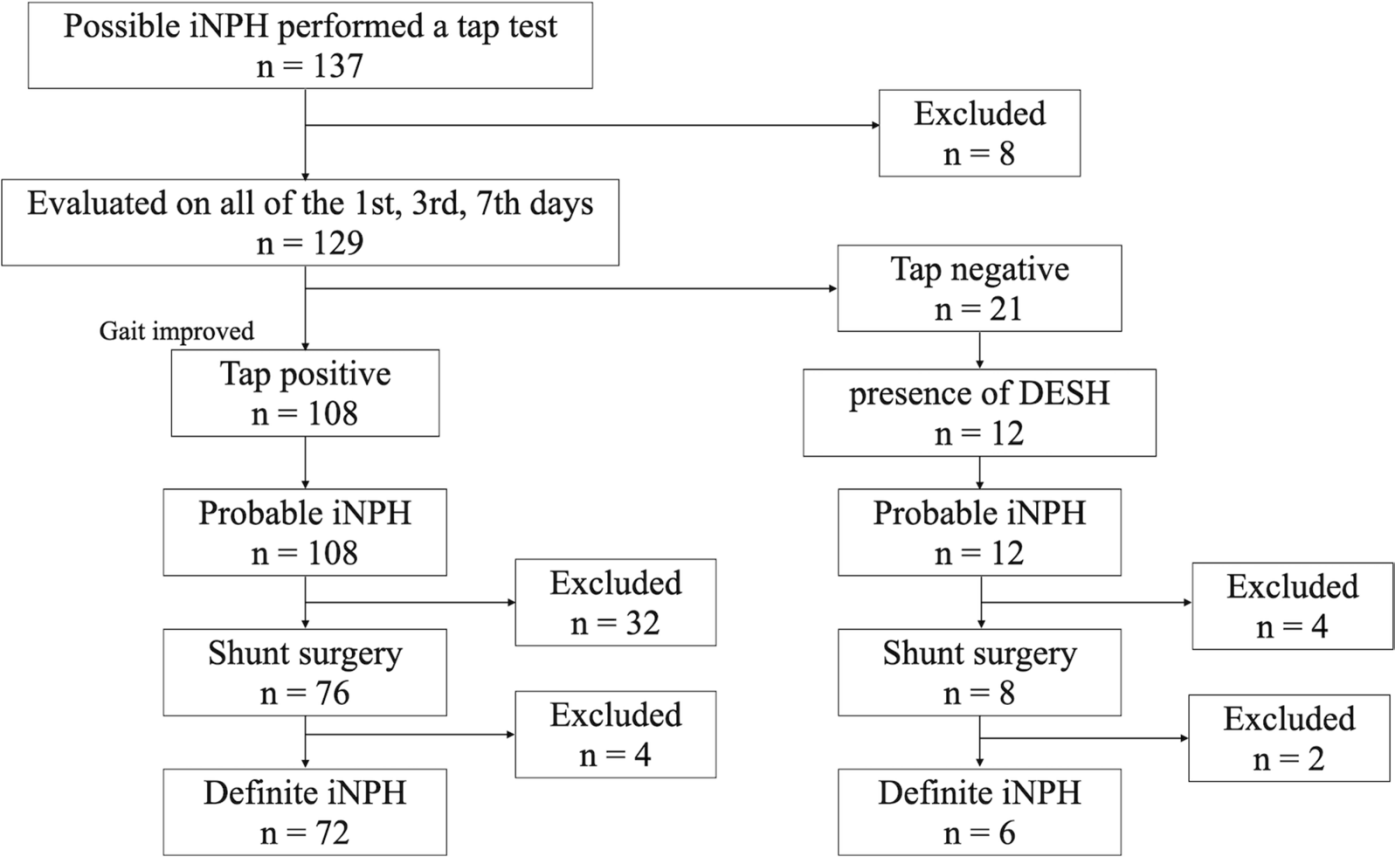
Flowchart of the process to identify probable idiopathic normal pressure hydrocephalus (iNPH) and definite iNPH in 137 possible iNPH patients performed tap tests.  Out of 137 possible iNPH patients who performed tap tests, 129 were evaluated for their gait function on all the 1st, the 3rd, and the 7th days after the tests. Among these 129 cases, 108 showed gait improvement on either the 1st, the 3rd, or 7th days, and they were diagnosed with probable iNPH. Twelve patients with DESH’s image findings were also diagnosed with probable iNPH but did not improve their gait function on any of the 1st, 3rd, or 7th days. Out of 120 probable iNPH patients, 84 underwent shunt surgery, and 78 were diagnosed with definite iNPH

Out of the 129 possible iNPH patients whose gait function was evaluated on the 1st, 3rd, and 7th days after tap tests, 57 patients showed the improvement of the gait function on the 1st day, 85 showed on any of the 1st or the 3rd days, and 108 showed on any of the 1st, the 3rd, or the 7th days. In other words, 57 patients were diagnosed with probable iNPH on the 1st day, 28 were newly on the 3rd, and 23 were newly on the 7th. In terms of the TUG time, among the 57 probable iNPH patients evaluated on the 1st day, 41 patients demonstrated an improvement of 5 s or more in the TUG, while the remaining 16 patients showed an improvement of 10 points or more in the iTUG score. For the 28 probable iNPH patients evaluated on the 3rd day, 20 patients exhibited an improvement of 5 s or more in the TUG, and the remaining 8 patients showed improvement in the iTUG score. Of the 23 probable iNPH patients diagnosed on the 7th day, 17 showed an improvement of 5 s or more in the TUG, and the remaining 6 showed improvement in the iTUG score. The number of probable iNPH patients whose gait function was evaluated on the 1st, 3rd, and the 7th days after tap tests (108 out of 129) was significantly larger than the number of those on the 1st and the 3rd days (85 out of 129) (*p* < 0.0001). The changes of the TUG time of each group are shown in Fig. 2. Apart from that, 12 patients of the 129 possible iNPH had the image findings of DESH and were diagnosed with probable iNPH (Fig. 1), while they did not improve the time of the TUG on any of the 1st, 3rd, and 7th days after the tap test Fig. 3.

### Proportion of definite iNPH

According to the above protocol, 120 patients were diagnosed with probable iNPH, 84 of whom underwent a shunt surgery (LPS, 51; VPS, 31; VAS, 2). Out of these 84 patients, 78 patients (92.9%) were identified as having definite iNPH with postoperative gait improvement. Six of these 78 definite iNPH patients did not show significant gait improvement on any of the 1st, 3rd, and 7th days after the tap test but had the image findings of DESH. Thirty-six of the 120 probable iNPH patients did not get the surgery; 24 were reluctant to select a surgical treatment with only minor symptoms, eight were too severe in cognitive impairment, and four had previously diagnosed Parkinson’s disease, which was supposed to be progressive and incurable. Among the patients who had a positive tap test result for the first time on each of the evaluation days, the number who underwent a shunt surgery was 48 out of 57 (84.2%) from the 1st day, 25 out of 28 (89.3%) from the 3rd, and 18 out of 23 (78.3%) from the 7th respectively. Of those post-surgical patients, the number who had improvement of symptoms and were eventually diagnosed with definite iNPH was correspondingly 42 (32.6%) from the 1st day, 19 (14.7%) from the 3rd, and 11 (8.5%) on the 7th (Table 2). This number of definite iNPH patients got significantly larger when the evaluation after the tap tests were performed on all of the 1st, the 3rd, and the 7th days (72 patients) than just on the 1st day (42 patients), (*p* = 0.00016) or both of the 1st and the 3rd day (61 patients) (*p* = 0.00074) (Table 2).

**Table 2:** Number of patients who showed gait improvement up to each evaluation day

Diagnosis	1st day (*n* = 129)	3rd day (*n* = 129)	7th day (*n* = 129)
Probable iNPH^a^, n	57	85	108
Shunt surgery^b^, n	48	63	84
Definite iNPH^c^, n	42	61	72

*Abbreviation:* *iNPH *idiopathic normal pressure hydrocephalus

^a^The patients who showed gait improvement after a tap test

^b^The patients who underwent a shunt surgery

^c^The patients who showed gait improvement after the shunt surgery

### Comparison of the background characteristics

There was no significant difference in the age, sex, the pre-tap test mRS, the pre-tap test TUG, and each of the three contents of the pre-tap test iNPH GS, between the two groups with improved gait function seen on at least the 1st or the 3rd days after the tap test, and seen only on the 7th day. On the other hand, only the pre-tap test MMSE showed higher scores in the former group than the latter (*p* = 0.044) (Table 1). There was no significant difference between the two groups concerning the EI, the CA, the size of the temporal horn, and the presence of the DESH (Table 1).

## Discussion

This study was to assess the necessity of checking the gait function on the 7th day after a tap. Consequently, we found that more patients showed gait improvement when we evaluated the gait function on the 1st, the 3rd, and the 7th days after tap tests than on the 1st or the 3rd day.

### Necessity of gait evaluation on the 7th day

There have been no published studies demonstrating the results of evaluating the gait on the 7th day. Of the 23 patients whose gait function was improved for the first time on the 7th day after tap tests, 11 patients (47.8%) were diagnosed with definite iNPH. This means that 11 (15.3%) of the overall 72 definite iNPH patients could have missed the opportunity of treatment if they had not been evaluated on the 7th day. This delayed response to a tap test could be caused by persistent epidural CSF leakage from the small dural hole after a spinal tap needle withdrawal. Huq et al. reported cases of persistent low CSF pressure after 7–14 days or longer after lumbar puncture [15].

### Characteristics of the group with gait improvement on the 7th day after tap tests

We compared the background characteristics between the group that showed gait improvement on either the 1st or the 3rd day after a tap test and the group that did it for the first time on the 7th day. There was no statistically significant difference except for the clinically minor gap in the pre-tap test MMSE.

### Limitations

This study has some limitations. First, this retrospective study was conducted at a single institution with a relatively small cohort. Further validation is needed with a more reliable study model. Second, although we showed the possible necessity of assessing the TUG tests until the 7th day after tap tests, it does not mean that an even longer follow-up is unrequired. We may need to consider extending the evaluation period for patients with no gait improvement until the 7th day so that they do not miss the chance of treatment. Third, among the 120 probable iNPH patients, 36 did not undertake the surgery. Our result may not represent the general nature of patients with positive tap tests. Fourth, we did not evaluate the gait on the 2nd day while there is a previous report that showed the maximum improvement in gait velocity [6].

## Conclusion

Gait evaluation on the 7th day after tap tests in addition to the first few days may reduce the iNPH patients who miss the opportunity of getting beneficial treatment.

## Data Availability

The datasets used and/or analyzed during the current study available from the corresponding author on reasonable request.
